# Long non-coding RNA ZFAS1 sponges miR-486 to promote osteosarcoma cells progression and metastasis *in vitro* and *vivo*

**DOI:** 10.18632/oncotarget.22032

**Published:** 2017-10-24

**Authors:** Nan Li, Zhen-Hui Sun, Min Fang, Jing-Yi Xin, Chun-You Wan

**Affiliations:** ^1^ Department of Traumatic Orthopedics, Tianjin Hospital, Tianjin 300211, China

**Keywords:** osteosarcoma, lncRNA, ZFAS1, miR-486, competing endogenous RNA

## Abstract

Long noncoding RNAs (lncRNAs) have been wildly demonstrated to participate in the osteosarcoma tumorigenesis. ZFAS1 is a novel identified lncRNA, however, its role in osteosarcoma is still unclear. In present study, we utilize lncRNA microarray assay to screen the lncRNA expression profile in osteosarcoma tissue, and investigate the regulatory function of ZFAS1 in osteosarcoma. LncRNA microarray assay revealed that lncRNA ZFAS1 was significantly up-regulated in 3 pairs of osteosarcoma and adjacent non-tumor tissue, which was confirmed by RT-PCR. Furthermore, in 53 pairs of osteosarcoma patient samples, the up-regulated expression of ZFAS1 was closely related to poor prognosis. *In vitro*, loss-of-function experiments showed that ZFAS1 knockdown significantly suppressed the proliferation, induced cycle arrest at G0/G1 phase and enhance apoptosis. *In vivo*, ZFAS1 knockdown inhibited the tumor growth. Bioinformatics online programs predicted that ZFAS1 sponge miR-486 at 3’-UTR with complementary binding sites, which was validated using luciferase reporter assay and RNA immunoprecipitation (RIP) assay. Rescue experiments confirmed that miR-486 could reverse the functions of ZFAS1 on osteosarcoma genesis. In conclusion, our results demonstrate that ZFAS1 act as competing endogenous RNA (ceRNA) for miR-486, and act as oncogene in osteosarcoma tumorigenesis, and discover the functional regulatory pathway of ZFAS1 sponging miR-486.

## INTRODUCTION

Osteosarcoma is a common primary bone tumor, accounting for the most frequent malignant bone tumor in children and adolescents [1, 2]. With the development of combinatorial chemotherapy, the prognosis of osteosarcoma patients has been significantly improved in the nearest statistics with nearly 60-70% of 5-year survival rate [3]. The major reason for the difficulty for therapy of osteosarcoma is the chemotherapy resistance of tumor cells, causing the unsatisfactory effect of osteosarcoma patients [4]. Moreover, the distant metastasis or local relapse after the surgical resection of primary tumor is another crucial risk factor for poor prognosis [5]. Thus, the effective therapeutic methods to suppress the metastasis and proliferation are the key for successful treatment.

Long non-coding RNAs (lncRNAs) are a type of new identified non-coding RNAs (ncRNAs) with more than 200 nucleotides in length. LncRNAs, acting as a vital member of family, have drawn the attention due to their increasing physiological function. The dysregulated lncRNAs expression has verified to contribute to series of human disease including cancers through regulating the chromatin remodeling, histone protein modification to affect functional gene expression. Besides, lncRNAs could function as microRNAs ‘sponge’, described as competing endogenous RNA (ceRNA), to adsorb the cytoplasm microRNAs, and then modulate the functional protein synthesis. For instance, in osteosarcoma pathogenesis, lncRNA PVT1 promotes osteosarcoma progression by acting as miR-195 sponge to regulate cell cycle arrest and apoptosis via miR-195 in osteosarcoma cells [6].

LncRNA zinc finger antisense 1 (ZFAS1) is an antisense transcript from 5’-end of Znfx1 gene, being firstly identified to play functional role in in patients with acute myocardial infarction [7, 8]. Moreover, the dysregulated ZFAS1 expression in cancer tissues have been discovered, for example breast cancer, gastric cancer and hepatocellular carcinoma [9, 10]. For instance, ZFAS1 is found to be overexpressed in gastric cancer and the increased level is associated with poor prognosis, besides, the oncogenic function is partly dependent on repressing KLF2 and NKD2 [11].

In conclusion, our data elucidated the regulatory function of lncRNA ZFAS1 in the development and progression of osteosarcoma via acting as competing endogenous RNA (ceRNA) of miR-486, suggesting an effective therapeutic strategy for osteosarcoma treatment.

## RESULTS

### LncRNA microarray revealed the expression profiles in osteosarcoma tissue

**Figure 1:**
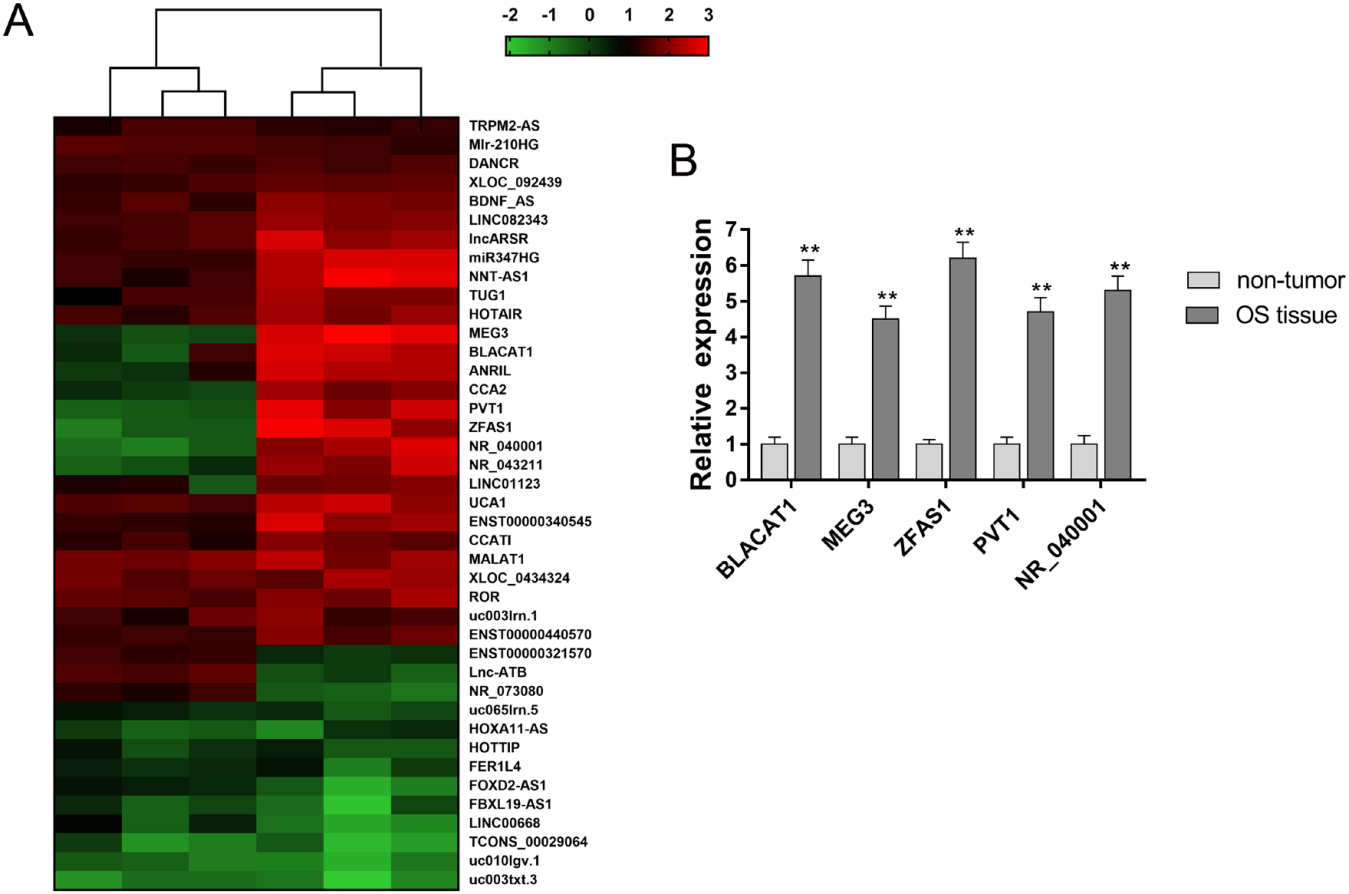
LncRNA microarray revealed the expression profiles in osteosarcoma tissue **(A)** Total 1,743 differently expressed lncRNA was screened using lncRNA microarray analysis. Heat map showed the 51 mostly significantly dysregulated lncRNA. **(B)** 5 up-regulated lncRNA was selected and measured using RT-PCR. Data are presented as the mean ± SD. *P<0.05, **P<0.01 compare to control group.

To investigate the lncRNA expression profiles of osteosarcoma tissue, we performed lncRNA microarray analysis for 3 pairs of osteosarcoma tissue and adjacent non-tumor tissue. Results showed that 1,743 differently expressed lncRNA was screened, and 51 mostly significantly dysregulated lncRNA was shown in heat map (Figure 1A). Then, 5 up-regulated lncRNA was selected from the candidate lncRNAs, whose expression was measured using RT-PCR (Figure 1B). Because the role of ZFAS1in osteosarcoma tumorigenesis was still unclear, thus, we chose ZFAS1 as the research target.

### LncRNA ZFAS1 was over-expressed in osteosarcoma tissue and associated with poor prognosis

**Figure 2:**
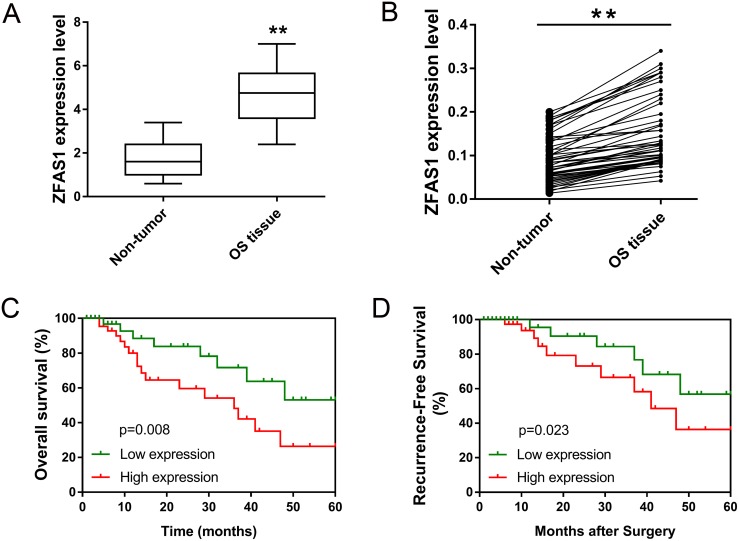
LncRNA ZFAS1 was over-expressed in osteosarcoma tissue and associated with poor prognosis **(A)** Expression of ZFAS1 in 53 cases of osteosarcoma tissue and adjacent non-tumor tissue detected by RT-PCR. **(B)** Corresponding match of ZFAS1 in 53 cases of osteosarcoma specimens within tumor tissue and adjacent non-tumor tissue. **(C)** Overall survival of osteosarcoma specimens with high and low expression of ZFAS1. **(D)** Recurrence-free survival of osteosarcoma specimens with high and low expression of ZFAS1. Data are presented as the mean ± SD. *P<0.05, **P<0.01 compare to control group.

LncRNA ZFAS1 had been reported to be up-regulated in breast cancer, gastric cancer and hepatocellular carcinoma [9, 10]. For osteosarcoma, we aimed to investigate the expression levels of ZFAS1 in osteosarcoma tissue from clinical samples. A total of 53 cases of osteosarcoma tissue were collected into present study. The relationship between ZFAS1 expression and clinicopathological characteristics of osteosarcoma patients was shown in Table 1. RT-PCR showed that ZFAS1 expression was significantly up-regulated in osteosarcoma tissue compared to that of in adjacent non-tumor tissue (Figure 2A). Specifically, among these specimens, total 48 cases were markedly up-regulated (Figure 2B). Afterwards, the expression levels of ZFAS1 were distinguished into high and low expression sub-group according to median value. To verify the interaction of ZFAS1 on osteosarcoma patient survival rate, we calculated the overall survival (OS) rate and recurrence-free survival (RFS) rate with post-surgical follow-up. Kaplan-Meier analysis and log-rank test showed that patients with high ZFAS1 expression had more poorer overall survivals than that with low expression levels (p=0.008) (Figure 2C). Besides, patients with high ZFAS1 expression levels had lower recurrence-free survival rate (p=0.023), being consistent with overall survivals (Figure 2D). Taken together, ZFAS1 expression was significantly up-regulated in osteosarcoma patients and predicted the poor prognosis, indicating the risk factor of ZFAS1 in osteosarcoma genesis.

**Table 1:** Relationship between ZFAS1 expression and clinicopathological characteristics of osteosarcoma patients

		n=53	ZFAS1		P
			low (N=21)	high (N=32)	
Gender	Male	33	12	21	0.574
	Female	20	9	11	
Age	<18	29	14	15	0.607
	≥18	24	7	17	
Tumor size (cm)	<8	22	13	9	0.026*
	≥8	31	8	23	
Enneking stage	I	17	10	7	0.092
	II	17	7	10	
	III	19	4	15	
Site	Femur/tibia	43	18	25	0.451
	Elsewhere	10	4	6	
Lung metastasis	No	37	18	19	0.019*
	Yes	16	4	12	
Recurrence	No	29	20	9	0.032*
	Yes	24	5	19	

*P<0.05 represents statistical differences.

### ZFAS1 knockdown suppressed the proliferation of osteosarcoma cells

**Figure 3:**
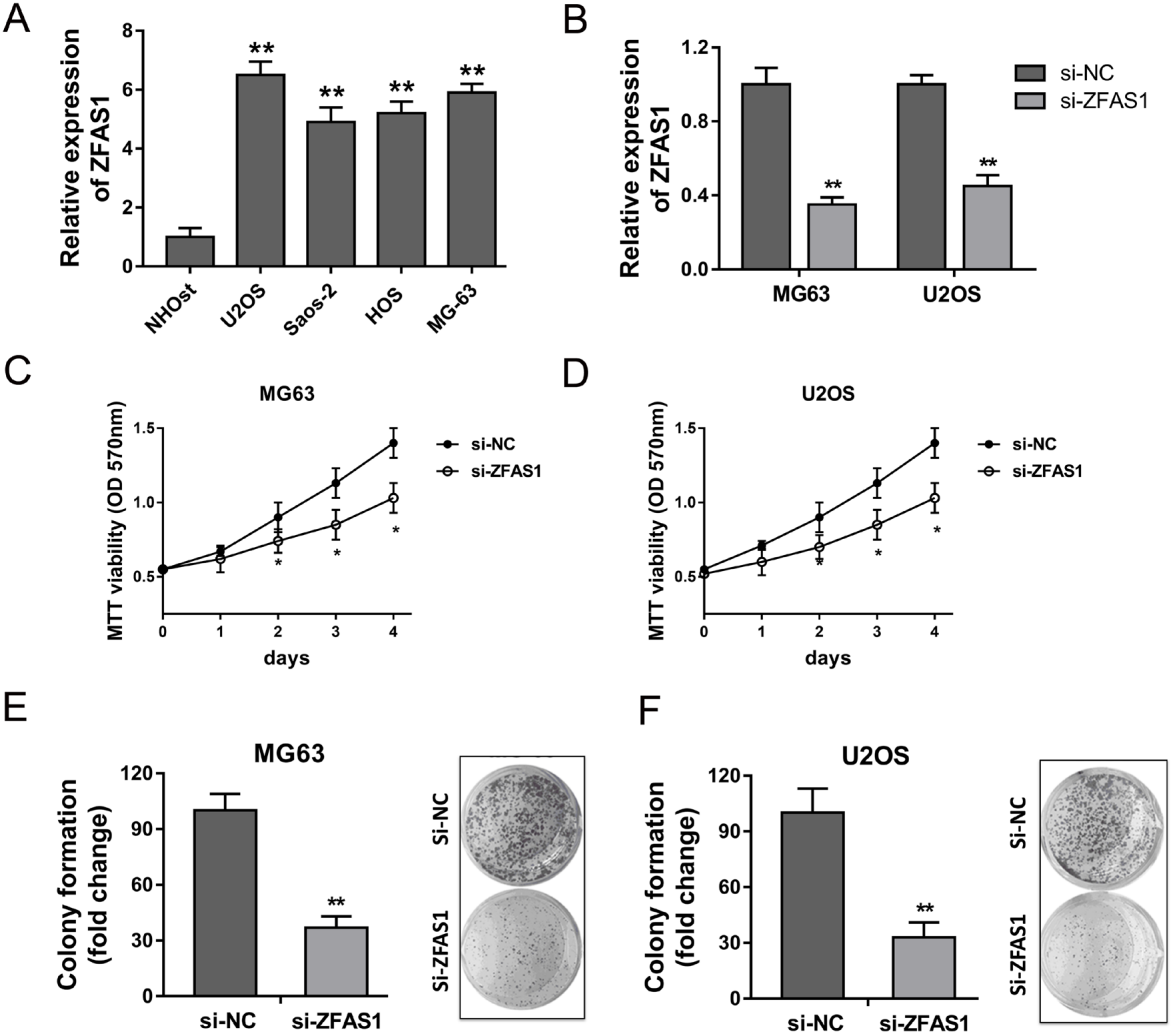
ZFAS1 knockdown suppressed the proliferation of osteosarcoma cells *in vitro* **(A)** Expression of ZFAS1 in osteosarcoma cell lines (U2OS, Saos-2, HOS and MG63) were detected by RT-PCR compared to normal human osteoplastic cell line (NHOst). **(B)** Knockdown of ZFAS1 expression transfected with synthetized small interfering RNAs in MG63 and U2OS cells. **(C, D)** MTT assay showed the proliferation ability of MG63 and U2OS cells. **(E, F)** Colony formation assay showed the relative clone formation number. Data are presented as the mean ± SD. *P<0.05, **P<0.01 compare to control group.

It had been testified that ZFAS1 was up-regulated in osteosarcoma tissue, and the high expression level of ZFAS1 was closely correlated with poor prognosis. Thus, we performed loss-of-functional experiments to test the function of ZFAS1 on proliferation of osteosarcoma cells *in vitro*. Firstly, RT-PCR showed that ZFAS1 expression in osteosarcoma cell lines (U2OS, Saos-2, HOS and MG63) were significantly up-regulated compared to normal human osteoplastic cell line (NHOst) (Figure 3A). The synthetized small interfering RNAs were respectively transfected into MG63 and U2OS cells to knock down the expression of ZFAS1 (Figure 3B). MTT vitality assay showed that ZFAS1 knockdown could suppress the proliferation ability of MG63 and U2OS cells (Figure 3C, 3D). Furthermore, colony formation assay showed that ZFAS1 knockdown could suppress the clone formation ability of MG63 and U2OS cells (Figure 3E, 3F). Taken together, loss-of-functional experiments revealed that ZFAS1 knockdown could inhibit the proliferation of osteosarcoma cells.

### ZFAS1 knockdown induced cell cycle arrest and promoted the apoptosis of osteosarcoma cells

**Figure 4:**
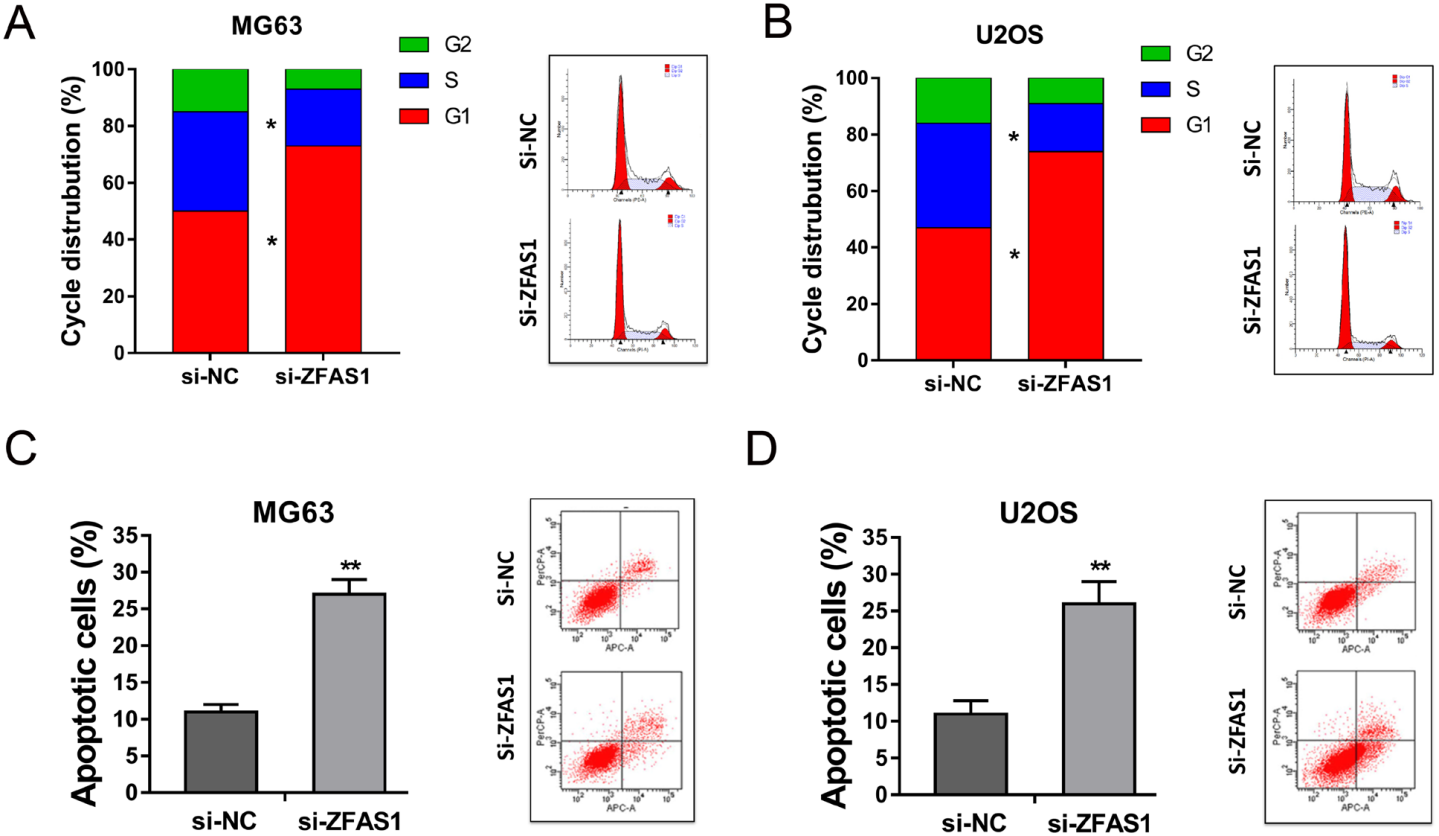
ZFAS1 knockdown induced cell cycle arrest and promoted the apoptosis of osteosarcoma cells **(A, B)** Flow cytometry showed the cell distribution of ZFAS1 knockdown group and control group in MG63 and U2OS. **(C, D)** Apoptosis of MG63 and U2OS cells transfected with si-ZFAS1 and control oligonucleotides. Data are presented as the mean ± SD. *P<0.05, **P<0.01 compare to control group.

For further investigation, we sequentially verified the role of ZFAS1 knockdown on apoptosis and cell cycle of osteosarcoma cells using flow cytometry. Results showed that MG63 and U2OS cells transfected with si-ZFAS1 induced cell cycle arrest at G0/G1 phase (Figure 4A, 4B). Besides, ZFAS1 knockdown promoted the apoptosis of MG63 and U2OS cells compared to si-NC group (Figure 4C, 4D). Thus, flow cytometry showed that ZFAS1 knockdown could induce cell cycle arrest and promote apoptosis on osteosarcoma cells *in vitro*.

### ZFAS1 knockdown suppressed the tumor growth *in vivo*

**Figure 5:**
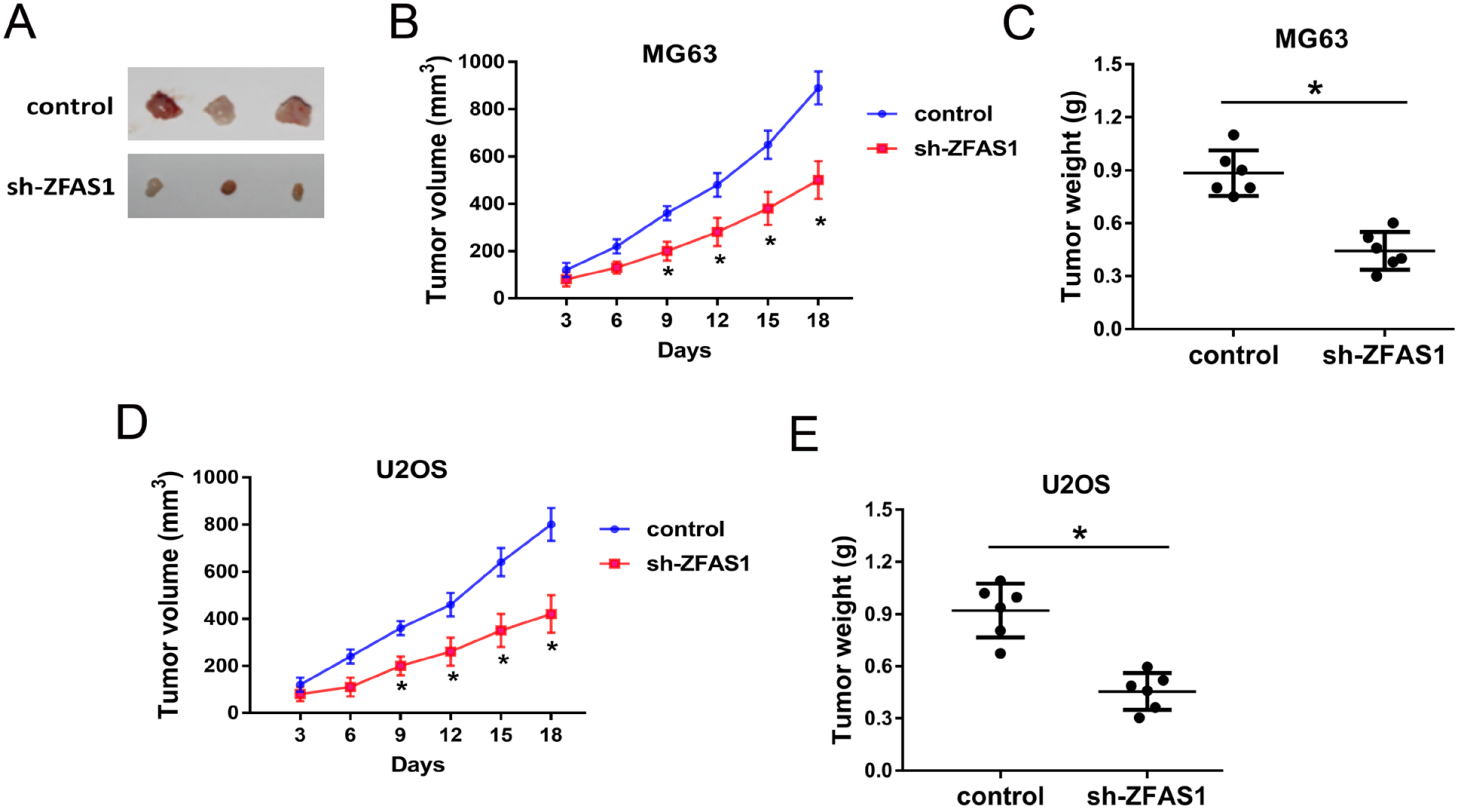
ZFAS1 knockdown suppressed the tumor growth *in vivo* **(A)** The resected tumors. The upper row was control group, and the lower row was sh-ZFAS1 group. **(B, C)** Injected with MG63 cells, tumour volumes measured every 3 days for three weeks. Tumour weights measured 3 weeks after injection and sacrifice. **(D, E)** Injected with U2OS cells, tumour volumes and weight were measured. Data are presented as the mean ± SD. *P<0.05 compare to control group.

To investigate the role of ZFAS1 on osteosarcoma tumorigenesis *in vivo*, xenograft assay was performed. MG63 and U2OS cells transfected with sh-ZFAS1 or control were subcutaneously injected into back of nude mice. After injection, the neoplastic weight and volume of sh-ZFAS1 group were substantially smaller compared to those of control group (Figure 5). Results of xenograft assay indicated that ZFAS1 knockdown could suppress the tumor growth *in vivo*.

### Bioinformatics prediction revealed the target miR-486 binding with ZFAS1 3’-UTR

**Figure 6:**
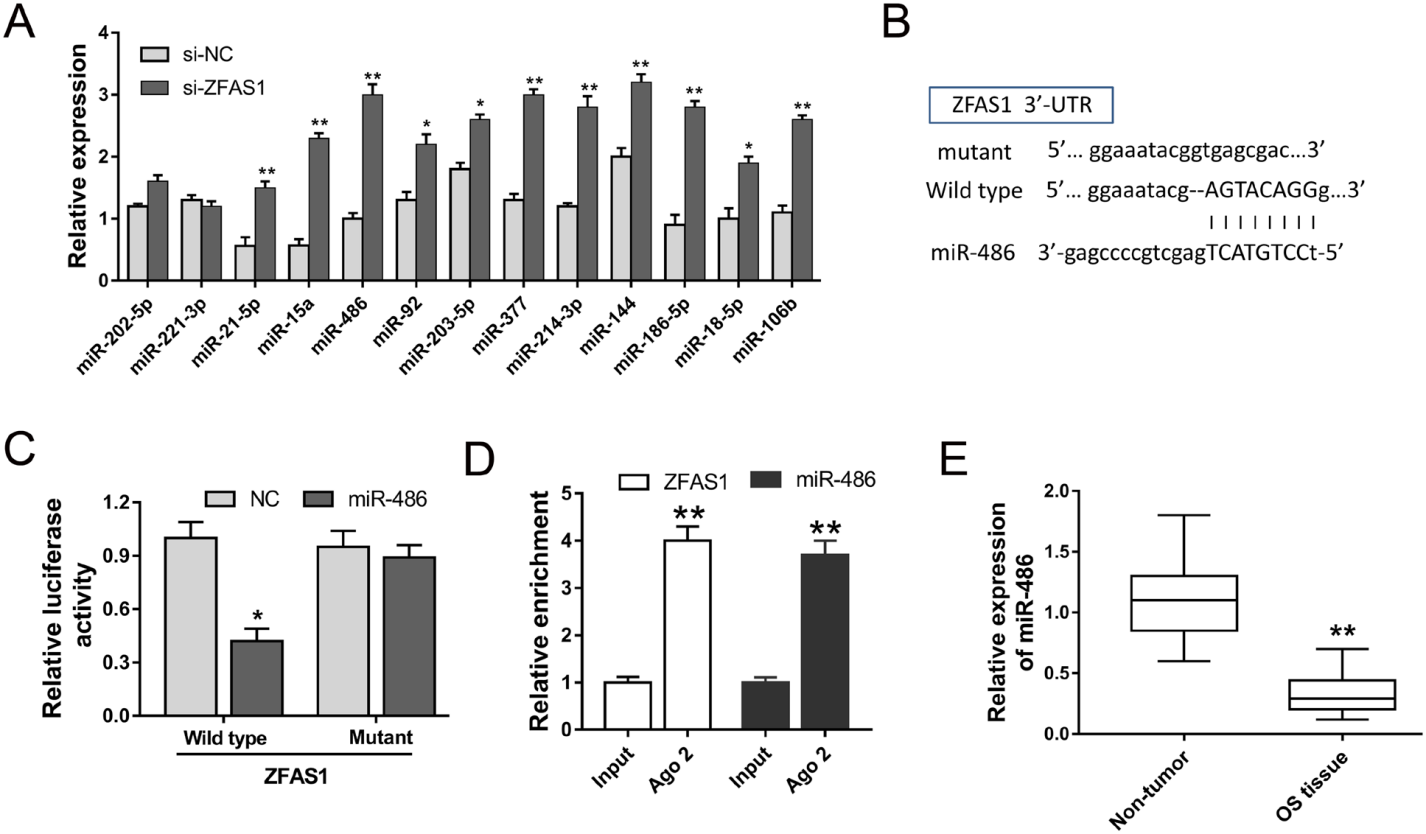
MiR-486 directly targeted ZFAS1 **(A)** Total 13 miRNAs were selected as candidate miRNAs. **(B)** The predicted complementary sequence between miR-486 and ZFAS1 3’-UTR. **(C)** Luciferase reporter assay showed the luciferase activity of HEK293K cells transfected with miR-486 mimics or negative control and ZFAS1 wild type or mutant. **(D)** RIP assay showed the enrichment of ZFAS1 and miR-486 Ago2-containing beads. **(E)** RT-PCR showed the expression of miR-486 in osteosarcoma tissue. Data are presented as the mean ± SD. *P<0.05, **P<0.01 compare to control group.

Although previous study had revealed that ZFAS1 acted as a risk factor in osteosarcoma tumorigenesis, the downstream regulatory pathway of ZFAS1 is still unclear. Bioinformatics prediction revealed that total 13 miRNAs might be the candidate miRNAs predicted by starBase website (http://starbase.sysu.edu.cn). Furthermore, RT-PCR was performed to test these candidate miRNAs expression levels in ZFAS1 knockdown MG-63 cells. As shown in Figure 6A, ZFAS1 silencing could significantly increase the expression levels of miRNAs. Furthermore, we chose the miR-486 and checked the complementary 3’-UTR sequence (Figure 6B). Then, the binding was validated using luciferase assay, revealing the high bound within ZFAS1 and miR-486 (Figure 6C). Besides, RNA immunoprecipitation (RIP) assay was performed to confirm the interaction between ZFAS1 and miR-486. RIP assay showed that ZFAS1 and miR-486 were significantly enriched in Ago2-containing beads compared to input group (Figure 6D). MiR-486 expression in osteosarcoma tissue was tested to be decreased (Figure 6E). Results demonstrated that miR-486 targeted ZFAS1 3’-UTR, which might be one of the downstream regulatory pathway of ZFAS1.

### ZFAS1 harbored miR-486 to regulate tumor progression of osteosarcoma cells

**Figure 7:**
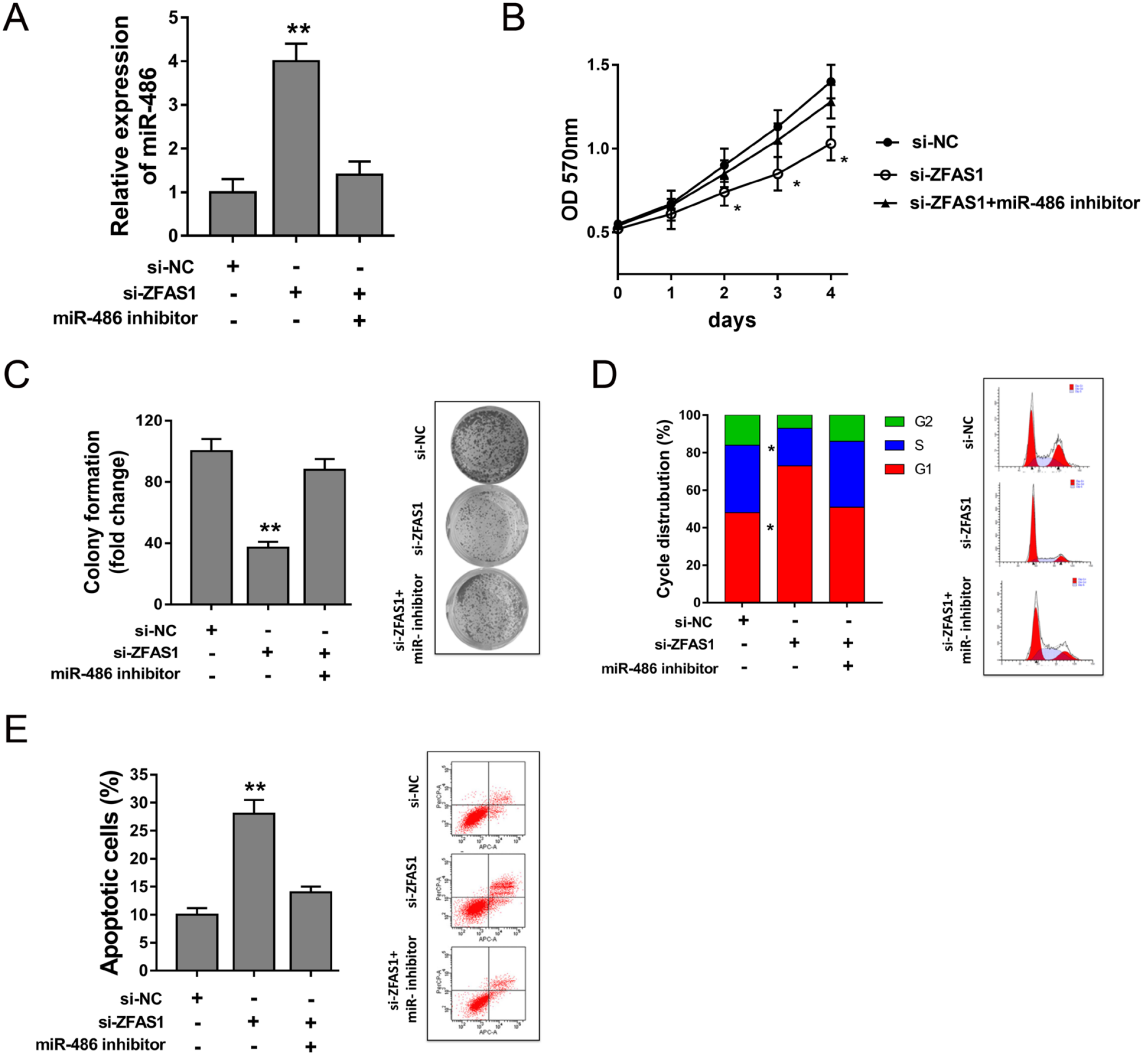
ZFAS1 harbored miR-486 to regulate tumor progression of osteosarcoma cells **(A)** Expression of miR-486 detected by RT-PCR in MG63 cells transfected with si-ZFAS1 and miR-486 inhibitor. **(B)** MTT assay revealed the proliferation of MMG63 cells. **(C)** Colony formation assay showed the clone number. **(D)** Cycle analysis revealed the cycle arrest at G0/G1 phase. **(E)** Apoptosis showed the apoptotic cell rate. Data are presented as the mean ± SD. *P<0.05, **P<0.01 compare to control group.

Previous study had revealed that miR-486 targeted ZFAS1 3’-UTR, thus, ZFAS1 might exert functional regulation via targeting miR-486. Rescue experiments were performed to verify the interaction in MG63 cells. ZFAS1 knockdown significantly increased the expression of miR-486, and miR-486 inhibitor could decrease the miR-486 expression induced by si-ZFAS1 (Figure 7A). MTT and colony formation assay showed that miR-486 inhibitor could reverse the inhibition of ZFAS1 on proliferation capacity (Figure 7B, 7C). Cell cycle analysis showed that miR-486 inhibitor alleviated the G0/G1 phase arrest compared to ZFAS1 knockdown (Figure 7D). Apoptosis assay showed that miR-486 inhibitor decreased the apoptotic cell induced by ZFAS1 knockdown (Figure 7E). In summary, rescue experiment revealed that miR-486 could rescue the function of ZFAS1 on osteosarcoma progression.

## DISCUSSION

In the past decades, accompany with the fast innovation and development of sequencing technique, many important progression have been achieved [12, 13]. High-throughput sequencing and bioinformatics analysis powerfully assist the non-coding RNA research on the epigenetic modification, discovering huge amounts of new identified functional lncRNAs in tumorigenesis [14]. Emerging evidences have demonstrated the important role of lncRNAs in series of tumorigenesis, as well as osteosarcoma [15].

Long noncoding RNAs (lncRNAs) are increasingly recognized to play important function on disease development and progression [16]. LncRNA ZFAS1 is an antisense transcript of the 5’ end of the protein-coding gene Znfx1. Besides, lncRNA ZFAS1 has been testified to be down-regulated in the breast tumors compared to normal tissue, and act as tumor biomarker in breast tumour pathology [17]. In rheumatoid arthritis (RA) patients, ZFAS1 expression was increased in synovial tissue and fibroblast-like synoviocytes (FLS) compared with that in healthy donors, and silence of ZFAS1 suppressed RA-FLS migration and invasion [18]. In present study, we detected the ZFAS1 expression in 53 cases of osteosarcoma patients and found the up-regulated expression of ZFAS1. Moreover, the over-expressed ZFAS1 was closely correlated with poor prognosis of osteosarcoma patients. In functional validation assay, ZFAS1 knockdown could significantly suppress the proliferation, induce cycle arrest at G0/G1 phase and promoted apoptotic cell rate. In summary, our study revealed the up-regulated expression of ZFAS1 in osteosarcoma tissue and cells, suggesting the tumor promoting factor of ZFAS1 in osteosarcoma genesis.

The importance of lncRNAs is emerging on occurrence and progression regulating covering the most of diseases, including cardiovascular system disease, hematological system disease and so on. For osteosarcoma genesis, increasing evidences have illustrated that lncRNAs are playing important roles in osteosarcoma carcinogenesis [19, 20]. Presently, the vital functions of series lncRNAs have been annotated and reported, for example MALAT1, HULC, H19 and HOTAIR [21–25]. In terms of ZFAS1, it has been testified to be oncogenic lncRNA in multiple cancers. For instance, ZFAS1 is significantly up-regulated in colorectal cancer tissue, and ZFAS1 silencing decreases tumor proliferation with G1-arrest via interacting with cyclin-dependent kinase 1 (CDK1)[9]. Liu G et al (2017) have reported that ZFAS1 overexpression is positively regulated malignant phenotypes by competitively binding the miR-200b and miR-200c and upregulating BMI1, and ZFAS1 also interacted with ZEB2 and regulated ZEB2 protein stability [26].

Admittedly, the most canonic regulating mode of lncRNAs is to act as molecular ‘sponge’ for miRNAs [27, 28]. In present study, we have uncovered the oncogenic role of ZFAS1 on osteosarcoma progression *in vitro*. To explore the downstream pathway of ZFAS1, we utilized bioinformatics approach to discover the potential miR-486 with complementary binding at ZFAS1 3’-UTR. Rescue experiments showed that miR-486 inhibitor could reverse the tumor-suppressing function of ZFAS1 knockdown on MG63 cells. In hepatocellular carcinoma, ZFAS1 functions as an oncogene in HCC progression by binding miR-150, which adsorbs ZEB1, MMP14 and MMP16 expression, and abrogating the tumor-suppressive [10]. For osteosarcoma, another lncRNA-ATB promotes proliferation, migration, and invasion depending on the regulation of miR-200s [29]. In osteosarcoma tissue, miR-486 was down-regulated, and further found that miR-486 inhibited the targeting of PKC-δ signaling pathways, and ulteriorly inhibit the growth and invasion of osteosarcoma cells [30]. In prostate cancer tissue and cells, miR-486-5p sponged Snail, a key regulator of the epithelial-mesenchymal transition, to suppress cell migration and the invasive ability [31].

Furthermore, increasing studies have discovered that lncRNAs can be stably found in tumor tissue or circulating peripheral blood [32–34]. The stable expression could raise the possibility that lncRNAs may serve as novel diagnostic biomarkers. Our study revealed the over-expression of ZFAS1 in osteosarcoma tissue and found the correlation of ZFAS1 up-regulation with poor prognosis, providing an effective diagnostic approach.

In conclusion, further researches on lncRNA regulation network are essential for understanding the molecular mechanism underlying osteosarcoma progression. Our present studies discover a novel regulatory pathway of ZFAS1 targeting miR-486 for diagnosis, prognosis, and therapy of osteosarcoma.

## MATERIALS AND METHODS

### Clinical patient samples

The study was approved by Ethics Committee and Institutional Review Board of Tianjin Hospital. All the research protocols had been examined by Tianjin Hospital and complied with the ethical statement. A total of 53 osteosarcoma patients were recruited in our study who undergo surgical operation at Tianjin Hospital from Apr 2015 to Dec 2016. None of them received radiotherapy or chemotherapy before surgery. Written informed consents had been obtained from all the enrolled patients. All the osteosarcoma tissues and paired adjacent non-tumor tissues were obtained from patients after surgical excision and were confirmed by two experienced pathologists. Then, the tissues were collected and frozen in liquid nitrogen until use.

### LncRNA microarray assay

Three pairs of osteosarcoma tissue and adjacent issue were used for lncRNA microarray analysis. Total RNA from each sample was quantified using the NanoDrop ND-1000 (Thermo Scientific, Wilmington, USA). Briefly, cDNA was reverse transcribed and lncRNA expression profile was performed using Human LncProfilers™ qPCR Array Kits (Biosciences, USA) according to manufacturer’s instruction.

### Osteosarcoma cell lines and culture

The human osteosarcoma cell lines (U2OS, Saos-2, HOS and MG-63) and normal human osteoplastic cell line (NHOst) were purchased from the American Type Culture Collection (ATCC, USA). Osteosarcoma cells were cultured in RPMI 1640 medium (Gibco, USA) added with 10% FBS and antibiotics (Gibco-BRL). HEK-293 cells were supplied by China Center for Type Culture Collection (CCTCC) and were cultured in Dulbecco modified Eagle medium (DMEM) contained 10% fetal bovine serum (FBS), 100 U/mL penicillin, and 100 ng/mL streptomycin. All cells were incubated at 37°C under a humidified atmosphere containing 5% CO_2_.

### Quantitative real-time PCR (qRT-PCR)

Total RNA was extracted using Trizol reagent (Invitrogen, Carlsbad, CA, USA) according to the manufacturer’s instructions. The cDNA was synthesized with the Reverse Transcription System (Promega, Madison, USA). RT-PCR was performed using the SYBR Green Master Mixture (Roche, America) reagent in ABI 7500 Real-time PCR instrument. U6 was acted as an internal control. Relative expression levels were calculated and normalized using the 2^−ΔΔCt^ method. All the primers were listed as follows: ZFAS1 forward, 5’-AAGCCACGTGCAGACATCTA-3’, reverse, 5’ CTACTTCCAACACCCGCATT-3’; miR-486, forward, 5’-TCATACTGTGGGAAACGCTT-3’, reverse 5’-GACACTCAGGGCAGGCAAA-3’; U6, forward, 5’-CGCTAGCACATATCGGCTA-3’, reverse, 5’-TTCTGCGACGAATTTGTCAT-3’.

### Oligonucleotides interfering transfection

Oligonucleotide sequences were designed and synthesized by GenePharma company (Shanghai, China). For transfection, siRNA was transfected into cells using Lipofectamine 2000 Reagent (Invitrogen, Carlsbad, Calif, USA) according to the manufacturer’s instructions.

### Cell proliferation assays

MTT assay and colony formation assay were performed to detect the proliferation. Cells were transfected siRNAs or mimics. After transfection for 48 hours, cells were seeded into 96-well plates at density of 5×10^3^ per well. Then indicated time points, cells per well were added 25 μl of 3-(4,5-Dimethylthiazol-2-yl)-2,5-diphenyl tetrazolium bromide (MTT, Promega, Madison, WI., USA) solution was added and incubated for 4 h. Absorbance was detected at 570 nm. For colony-forming growth assays, 2×10^3^ cells were added into six well plates with1640 medium containing 10% FBS. The cancer cells were calculated after 14 days of culture and counted by manually counted. All measurements were repeated three times in quadruplicates.

### Luciferase reporter assays

The HEK 293T cells were obtained from ATCC and cultured at density of 5×10^4^ cells/well in 24-well plates. For luciferase assay, the full-length 3’-UTR of ZFAS1 containing miR-486 binding sites was cloned into the downstream of the firefly luciferase gene in pGL3 (Invitrogen) to establish pGL3-luc-ZFAS1 wild type and mutant. The co-transfection of plasmid and oligonucleotide sequence were performed using Lipofectamine 3000 (Invitrogen) according to the manufacturer’s instructions. The luciferase activities were measured using a dual-luciferase reporter assay system (Promega, Madison, WI).

### RNA immunoprecipitation (RIP) assay

RNA immunoprecipitation (RIP) assays were performed to confirm the interaction of ZFAS1 and miR-486. In brief, RIP was performed using EZMagna RIP RNA-binding protein immunoprecipitation kit (Millipore, Billerica, MA, USA) according to the manufacturer’s instructions. Cells in different groups were lysed using RNA lysis buffer containing protease inhibitor and RNase inhibitor. Cells were incubated with the RIP buffer containing magnetic beads coated with Ago2 antibodies (Millipore). IgG acted as a negative control (input group). After incubation for 2 h at 4°C, coprecipitated RNAs were isolated and measured by PCR analysis.

### Cell apoptosis and cell cycle assay

Flow cytometry was performed for cell apoptosis assay. In brief, cells were plated into 6-well plates at density of 1x10^5^ cells/well. After siRNA transfection, cells were harvested by trypsinization and washed with PBS. Then, cells were resuspended in Annexin-binding buffer, and then 5 μl Annexin V-FITC and 1 μl PI were appended. Afterwards, 5 μl Annexin V-FITC and 1 μl PI double-staining (BD Biosciences, Franklin Lakes, NJ, USA) was used. The apoptotic cells were performed using flow cytometric analyzes (Attune, Darmstadt, Germany) and the Flowjo software (Tree Star Corp, Ashland, OR) was used to analyze the results. All the tests were performed in triplicate. For cell cycle analysis, U2OS and MG-63 cells were resuspended using PBS containing 70% ethanol and propidium iodide (0.1 mg/ml). Cell cycle was analyzed by flow cytometer. Images were obtained from the FCM. All the tests were performed in triplicate.

### Xenograft nude mouse model

Male BALB/c nude mice (6 weeks) were maintained in clean conditions and used for xenograft assay. MG63 and U2OS cells (4×10^5^ per 100 ml) were respectively transfected with shRNAs of ZFAS1 or control, and then subcutaneously injected into back of nude mice. After that, tumour size was measured every 4 days, and tumor weight was measured after sacrifice of mice. The xenograft mice assay was approved by the Committee on Animal Welfare of Tianjin Hospital.

### Statistical analysis

All data were represented as mean ±standard deviation and statistical analyses were performed using SPSS (19.0 vision, Chicago, IL, USA). The differences were evaluated by the Student t-test and one way ANOVA. Kaplan–Meier methods with the log-rank test were performed to calculate disease free survival and overall survival. The significant difference was set as 0.05.
